# A Cross-Sectional Study of the Marital Attitudes of Pregnant Women at Risk for Cystic Fibrosis and Psychological Impact of Prenatal Screening

**DOI:** 10.3390/ijerph19148698

**Published:** 2022-07-17

**Authors:** Zoran Laurentiu Popa, Madalin-Marius Margan, Izabella Petre, Elena Bernad, Lavinia Stelea, Veronica Daniela Chiriac, Marius Craina, Ioana Mihaela Ciuca, Anca Mihaela Bina

**Affiliations:** 1 Department of Obstetrics and Gynecology, “Victor Babes” University of Medicine and Pharmacy Timisoara, 300041 Timisoara, Romania; popa.zoran@umft.ro (Z.L.P.); petre.izabella@umft.ro (I.P.); bernad.elena@umft.ro (E.B.); stelea.lavinia@umft.ro (L.S.); chiriac.veronica@umft.ro (V.D.C.); craina.marius@umft.ro (M.C.); 2 Centre for Translational Research and Systems Medicine, “Victor Babes” University of Medicine and Pharmacy Timisoara, 300041 Timisoara, Romania; lungu.anca@umft.ro; 3 Department of Microscopic Morphology, “Victor Babes” University of Medicine and Pharmacy Timisoara, 300041 Timisoara, Romania; 4 Department of Pediatrics, “Victor Babes” University of Medicine and Pharmacy Timisoara, 300041 Timisoara, Romania; ciuca.ioana@umft.ro; 5 Department III Functional Sciences, Discipline Pathophysiology, “Victor Babes” University of Medicine and Pharmacy Timisoara, 300041 Timisoara, Romania

**Keywords:** cystic fibrosis, mucoviscidosis, prenatal screening, mental health, marital attitudes

## Abstract

Cystic fibrosis (CF) is one of the most frequent genetic disorders in those with Northern European ancestry. Prenatal testing for cystic fibrosis may be used to plan and prepare for the birth of a child with the disease or to determine whether to terminate the pregnancy. The accessibility of prenatal detection for women with a high genetic risk of delivering a child with cystic fibrosis is determined by CF carriers and those affected by the disease. Moreover, prenatal testing for CF is mainly dependent on invasive diagnostic tests that can influence the mental health of the pregnant woman, and it is assumed that the birth of a CF child will have a serious influence on the couple’s subsequent family planning and marital behavior. The purpose of this research was to examine the marital attitudes of women at risk for cystic fibrosis and the psychological effect of screening for CF among pregnant women. The study followed a cross-sectional design with five questionnaires comprising Prenatal Attachment Interview (PAI), Maternal Antenatal Attachment Scale (MAAS), Pregnancy-Related Anxiety Questionnaire (PRAQ-R2), the Prenatal Psychosocial Profile (PPP), and the Marital Intimacy Questionnaire (MIQ). A total of 84 pregnant women were included in the “carriers” group for CFTR and 91 in the “non-carrier” group. CFTR-carrier mothers were likely to be more affectionate to the fetus, with better maternal–fetal quality and intensity of attachment. The same group of pregnant women was less scared of giving birth or worried about bearing a physically or mentally handicapped child compared to women who were expecting the prenatal diagnosis test for being at risk of delivering a newborn with malformations. CFTR-carrier pregnant women did not score significantly different results in the Prenatal Psychosocial Profile regarding stress levels, social support, and self-esteem. It was also found that intimacy and consensus problems inside the marriage were significantly more often experienced by CFTR carriers. Based on the current findings, it is likely that CFTR-carrier mothers have a better perception of the possible pregnancy outcomes by knowing their abnormal gene carrier status. Therefore, the psychological impact of invasive diagnostic tests is lower in this category compared with those who are unaware of the possible pregnancy outcomes. However, we promote a future analysis for pregnant women with moderate risk of giving birth to a child with single-gene mutations such as cystic fibrosis or other congenital malformations that undergo noninvasive prenatal diagnosis tests, as they become more accurate and might cause lower pre-diagnosis stress levels.

## 1. Introduction

Cystic fibrosis (CF), identified as OMIM 219700, is the most prevalent hereditary condition in Caucasian populations, affecting one in every 2500–3500 live births, with a carrier prevalence of one in every 25–30 persons [1], which is caused by a monogenic autosomal recessive mutation in the CF transmembrane conductance regulator (CFTR) gene [2]. Even though the gradually worsening pulmonary disease is the leading cause of morbidity and mortality in these patients, the disorder is also characterized by pancreatic exocrine insufficiency, meconium ileus, liver disease, congenital bilateral absence of the vas deferens in males, and elevated sweat chloride concentration levels in its classical form [3,4]. Furthermore, the vast clinical spectrum correlated with CFTR gene mutations varies from severe classical CF with pancreatic insufficiency to delayed CFTR-related diseases such as bronchiectasis or male sterility, which is caused by a congenital bilateral absence of the vas deferens [5]. More than 2000 CFTR variations have been found; although the ACMG guidelines are used in the majority of illnesses, the CF community has maintained two classifications: a functional classification dedicated to the selection of a genotype-based treatment and a clinical classification permitting pregestational genetic counseling [6,7].

Among the many responsibilities of an obstetrician is to provide an efficient and fast diagnosis of potential pregnancy complications and genetic abnormalities since this is the first step in the care of the patient. Prior to genetic testing, accurate newborn screening and fast identification of CF were unattainable for the majority of individuals [8]. In reality, children with CF frequently endured a diagnostic odyssey and suffered from permanent malnutrition or lung illness before sweat chloride testing confirmed their diagnosis [9,10]. It is estimated that many died without a diagnosis, while the causes of death might have been hyponatremic/hypochloremic dehydration, protein–energy malnutrition, or catastrophic lung illness [11]. These deaths are expected to continue in places of the globe where CF screening has not been adopted [12]. The benefits of early diagnosis are not only apparent but have also been recognized in research demonstrating that survival is significantly higher when a patient is diagnosed before the age of three months [13]. In those without a family background of CF, it might be detected during the prenatal period in the context of ultrasonography with digestive abnormalities such as fetal hyperechogenic bowel and intestinal loop dilatation, most commonly throughout the second trimester when 0.5% to 9.9% of people with hyperechogenic bowel are diagnosed with CF [14,15].

The need for prenatal diagnosis for hereditary genetic illnesses is expanding substantially due to the growing frequency of identifiable disease genes and the improvement of diagnosis methods. Noninvasive prenatal diagnosis may be used to detect aneuploidies and microdeletions, but only in autosomal recessive disorders with distinct parental mutations [16] can it be used to diagnose hereditary diseases. Consequently, the majority of prenatal diagnoses for inherited genetic illnesses are accomplished by analyzing DNA from amniocytes collected between the 15th and 20th week of gestation or from chorionic villi collected during the first trimester ranging from the 10th to 12th week of gestation [17].

Prenatal screening for CF offers the expectant mother and her spouse an estimate of their child’s potential abnormality risk. The pair must then decide whether to undertake further, more invasive diagnostic procedures depending on this chance or to terminate the pregnancy. The couple experiences greater stress and tension throughout this decision-making process [18], while other studies have determined that, on its own, disclosing susceptibility to give birth to a child with abnormalities carries a certain psychological impact [19]. Anxiety over the child’s medical history and a lack of dispositional optimism predicted anxiety later in the testing procedure. Even in women with normal test results, prenatal testing for genetic abnormalities is related with a moderate degree of emotional disturbance that diminishes but does not entirely disappear after testing [20,21,22]. The levels of stress and anxiety may be affected by the information presented, the counseling and psychological support offered, the prenatal test findings, and the outcome of the pregnancy. It is likely that sufficient care and support may favorably improve fetal–maternal bonding by reducing maternal stress and anxiety levels. There is growing evidence that prenatal anxiety and stress may have long-term consequences for both the pregnant mother and her baby, as well as cause relational issues and adaptive behaviors [23,24]. Therefore, it is crucial to study prenatal anxiety and stress. The current study aimed to examine the marital attitudes of pregnant women at risk for CF and the psychological effect of screening for CF among pregnant women.

## 2. Materials and Methods

### 2.1. Study Design and Participants

From 9 December 2021 to 9 May 2022, a cross-sectional study was conducted on pregnant outpatients and their spouses at the University Clinic of Obstetrics and Gynecology “Bega” associated with the University of Medicine and Pharmacy in Timisoara, Romania. Patients were told of the purpose and consequences of the research, and each patient signed a written informed consent form to be included in the current study. The surveys were delivered online in accordance with COVID-19 pandemic restrictions, and data collection was performed based on the complete answers received in parallel with paper records of the pregnant women followed at our clinic. Pregnant patients with a documented status of CF gene carrier (CFTR) were included in the study. Incomplete questionnaires, lack of patient consent, and incomplete paper records were considered exclusion criteria. Our research was done in accordance with the Helsinki Declaration Guidelines for scientific studies involving human participants, and it was authorized by the Scientific Ethics Committee of the Timisoara Municipal Hospital on 23 February 2021, code I-32467/23.12.2021).

### 2.2. Surveys and Variables

A convenience sampling method to calculate the appropriate sample size was employed for the group of pregnant women with CFTR gene mutation. It was estimated to comprise at least 60 pregnant women, with a margin of error of 5% at a confidence level of 95% and an assumed frequency of 4–5% in the general population, considering our hospital serves a region with approximately 1 million inhabitants [25]. Out of the 108 CFTR-carrier pregnant women who were requested to participate, 93 consented to participate in the research and fill out our questionnaires, while nine others failed to provide consistent and complete answers, leaving a total of 84 validated questionnaires in the “carriers” group. A comparison group was established from pregnant women without CFTR gene mutation who were planned for invasive prenatal diagnosis due to various reasons other than CF. The same convenience sampling method was employed to calculate the sample size for the comparison group, which, according to our clinic records, consisted of around 3% of pregnancies being referred for invasive testing. They were requested to complete the same questionnaires. Of 100 surveyed patients, a total of 91 were successfully included in the “non-carrier” group. The time of surveillance was between two and three weeks before the scheduled invasive diagnostic test.

The survey collected information on the demographics, obstetrical characteristics, depression, anxiety, quality of life, and coping mechanisms of the participants under stressful circumstances. Participants were requested to complete the following five standardized questionnaires: (1) Prenatal Attachment Interview (PAI); (2) Maternal Antenatal Attachment Scale (MAAS); (3) Pregnancy-Related Anxiety Questionnaire (PRAQ-R2), which includes the Fear of Bearing a Physically or Mentally Handicapped Child Subscale; (4) the Prenatal Psychosocial Profile (PPP), and (5) the Marital Intimacy Questionnaire (MIQ).

Using the PAI survey, on a 4-point scale, pregnant mothers rated on 21 questions how often they participated in particular thoughts or behaviors toward the fetus: 1 = nearly never, 2 = occasionally, 3 = frequently, and 4 = almost constantly, totaling a maximum of 84 points [26].

The MAAS questionnaire was selected because it emphasizes the mother’s sentiments and attitude regarding her fetus in a highly particular manner [27]. It consists of a 19-item questionnaire that examines attachment quality and attachment intensity. Scores above the mean (49.2) for quality of attachment are considered positive, but scores below the mean are considered ambivalent or distant. For the strength of attachment, scores over 26.5 are deemed positive, but scores below 26.5 are deemed uninvolved or ambivalently involved. A high global attachment score over 75.7 implies a strong bond with the fetus, with the mom being more devoted.

The PRAQ-R2 was evaluated using translations into Romanian. Each item’s score varied from 1 (certainly false) to 5 (definitely true). The survey items may be organized into three subscales. The first subscale, Fear of giving birth, consists of three statements, such as “I am anxious about the discomfort of contractions and labor pain.” The second subscale, Concerns about carrying a baby with a physical or mental disability, consists of four questions, including “I occasionally worry that our child will have bad health or be prone to disease.” The third subscale, Concern about one’s personal looks, consists of three questions, including “I am concerned about my rapid weight gain.” Total and factor sum scores were computed for the PRAQ. Items on the PRAQ-R ranged from 2 to 11 (total sum), and the factor sums were F1 (Fear of giving birth—summing the items 2, 6, and 8), F2 (Worries about carrying a physically or mentally disabled newborn—summing items 4, 9, 10, and 11), and F3 (Concern about personal looks—summing items 3, 5, and 7) [28].

The PPP is an effective clinical and research instrument that has proven to be a reliable measure for Caucasian and African American women. It employs a Likert-type scale with 44 questions to measure three components: stress, social support (split into spouse support and support from other people), and self-esteem [29].

The MIQ includes 56 questions rated on a 5-point Likert scale ranging from 1 (certainly not true) to 5 (certainly true). Five aspects of intimacy are measured by the MIQ: intimacy difficulties, agreement, transparency, love, and dedication. The greater the result on the subscale for intimacy issues, the worse the difficulty in that aspect. Higher scores on the remaining four subscales imply greater performance in each of the six intimacy domains. Except for the commitment subscale, the questionnaire exhibits adequate validity and internal consistency of more than 0.80 for all subscale scores [30].

### 2.3. Statistical Analysis

To perform descriptive and inferential statistics, we utilized the IBM SPSS Statistics for Windows, Version 27.0 (Armonk, NY, USA, IBM Corp.) and MS EXCEL (Microsoft Corp. Redmond, Washington, DC, USA). Mean and standard deviation were employed to describe continuous data, whilst absolute values and percentages were utilized to represent categorical variables. The Student’s *t*-test was used to compare the mean values of the data examined in this investigation. Chi-square and Fisher’s tests were used for proportional comparisons between the two research groups. The significance threshold was established at alpha = 0.05.

## 3. Results

### 3.1. Analysis of Background Data

The two comparison groups comprised 84 CFTR carrier pregnant women and 91 non-carrier pregnant women. The comparison of background characteristics presented in Table 1 indicated a statistically significant age difference between study groups. Among carriers, there were 39 (46.4%) in the 25–35 age range, compared with 41 (45.1%) in the age range higher than 35 years old (*p*-value = 0.002). Most of the respondents were residing in urban areas (60.7% vs. 56.0%) and more than 93% were living as married couples or concubines. Approximately half of all respondents were from the medium-income group, and 80% were employed, without significant differences between groups. The sexual behavior of the study participants consisted of 1–3 times per month in 52.4% in the carriers group, compared to 56.0% in the non-carriers group. Lastly, there was one woman who consumed alcohol frequently in the carriers group, compared to three in the non-carriers group. A total of 16.7% of women in the carrier group reported being frequent smokers, compared to 13.2% among non-carriers.

### 3.2. Analysis of Medical History

The obstetrical characteristics and comorbid conditions described in Table 2 showed a statistically significant difference in the proportion of gravidities, where 75.0% of CFTR-carrier pregnant women reported to be at the first pregnancy, compared with 61.5% in the other group (*p*-value = 0.045). There were no statistically significant differences in parity, number of pregnancy-associated complications, or comorbidities between groups. The most frequent type of invasive prenatal evaluation scheduled to be performed was amniocentesis, for 54.8% of carriers and 60.4% of non-carriers (*p*-value = 0.447). Approximately 75% of all respondents were in the normal weight body mass index range (18.5–24.9 kg/m^2^). There were six (7.1%) CFTR-carrier women with a history of depression, compared to eight (8.8%) in the non-carrier group. In total, it was observed that 16 (9.1%) patients had been infected with SARS-CoV-2 since the beginning of the COVID-19 pandemic.

### 3.3. Analysis of Questionnaires

**Figure 1:**
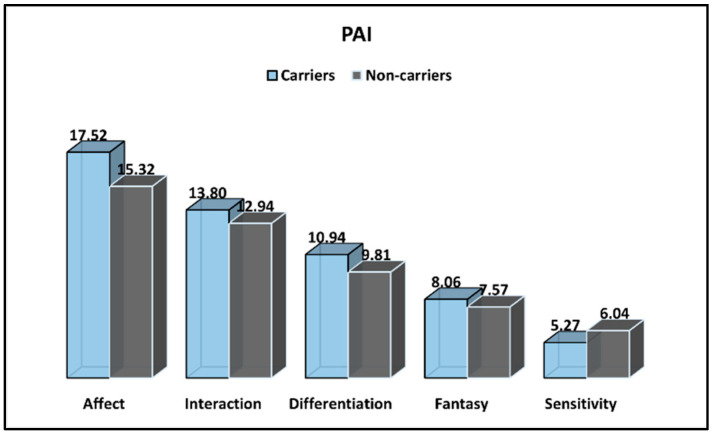
PAI survey results for CFTR-carrier and non-carrier pregnant women.

The Prenatal Attachment Interview (PAI) results for CFTR-carrier and non-carrier pregnant women are presented in Table 3 and Figure 1. It was observed that CFTR carriers were more affectionate than non-carrier pregnant women were (*p*-value = 0.002), and they showed higher differentiation scores (10.94 vs. 9.81, *p*-value = 0.033). However, sensitivity scores were significantly greater among non-carriers (6.04 vs. 5.27, *p*-value = 0.042). In general, the total PAI score was statistically higher among carriers (57.13 vs. 51.35, *p*-value < 0.001). The differentiation and fantasy average scores were not significantly different between groups.

**Figure 2:**
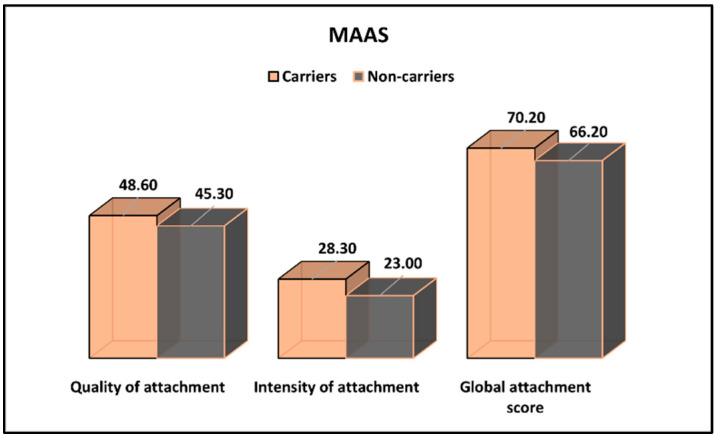
MAAS survey results for CFTR-carrier and non-carrier pregnant women.

The Maternal Antenatal Attachment Scale results for CFTR-carrier and non-carrier pregnant women are presented in Table 4 and Figure 2. It was observed that the quality of attachment and intensity of attachment were statistically significantly higher among CFTR carriers (48.6 vs. 45.3, *p*-value = 0.045 and 28.3 vs. 23.0, *p*-value < 0.001, respectively). However, the global attachment score was not significantly different between the study groups.

**Figure 3:**
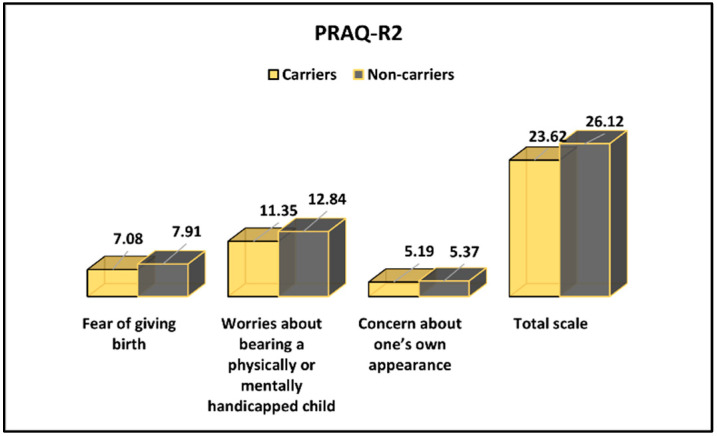
PRAQ-R2 survey results between CFTR-carrier and non-carrier pregnant women.

The Pregnancy-Related Anxiety Questionnaire results for CFTR-carrier and non-carrier pregnant women are presented in Table 5 and Figure 3. It was observed that Worries about bearing a physically or mentally handicapped child, and the PRAQ-R2 total score were significantly higher among CFTR non-carrier pregnant women (11.35 vs. 12.84, *p*-value = 0.011 and 23.62 vs. 26.12, *p*-value = 0.031, respectively). The scores reported for Fear of giving birth and Concerns about one’s own appearance were not different between groups.

**Figure 4:**
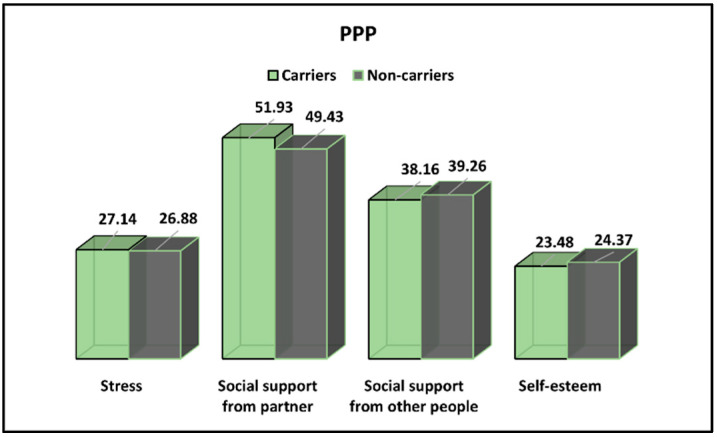
PPP survey results for CFTR-carrier and non-carrier pregnant women.

The Prenatal Psychosocial Profile results for CFTR-carrier and non-carrier pregnant women are presented in Table 6 and Figure 4. Stress levels were described not to be statistically higher among CFTR-carrier pregnant women scheduled for prenatal diagnosis (27.14 vs. 26.88, *p*-value = 0.724). There were also no significant differences in social support from other people and self-esteem scores as reported by both study groups.

**Figure 5:**
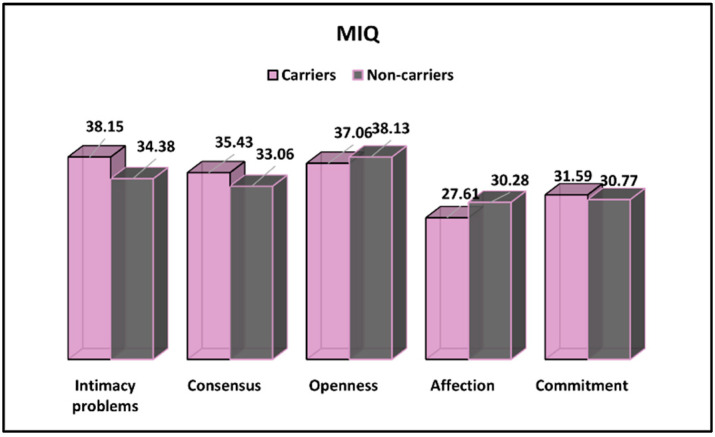
MIQ survey results for CFTR-carrier and non-carrier pregnant women.

Lastly, The Marital Intimacy Questionnaire results for CFTR-carrier and non-carrier pregnant women are described in Table 7 and Figure 5. It was observed that intimacy and consensus problems were more often experienced by CFTR carriers (38.15 vs. 34.38, *p*-value = 0.009 and 35.43 vs. 3.06, *p*-value = 0.039, respectively). On the other side, openness and affection levels were observed to be higher among non-carriers, with significant differences only regarding the affection levels.

## 4. Discussion

### 4.1. Supporting Literature

The current study determined a series of interesting findings in regards to the marital attitudes of pregnant women at risk for giving birth to a child with CF in correlation with the psychological impact of expecting the prenatal screening that will elucidate the diagnosis of the fetus. It was observed that CFTR-carrier mothers are likely to be more affectionate to the fetus, with better maternal–fetal quality and intensity of attachment. The same group of pregnant women were less scared of giving birth or worried about bearing a physically or mentally handicapped child compared to women who were expecting the prenatal diagnosis test for being at risk for delivering a newborn with malformations. On the other side, CFTR-positive pregnant women did not score significantly different results in the Prenatal Psychosocial Profile in regards to stress levels, social support, and self-esteem. Lastly, the current study evaluated marital intimacy issues of the participants and found that intimacy and consensus problems were more often experienced by CFTR carriers.

Pregnant women with CFTR gene mutations were observed in the current study to be less worried about bearing a physically or mentally handicapped child before the prenatal invasive diagnostic test, as compared with the other pregnant women at risk for giving birth to a child with genetic disorders or congenital malformations. Regarding the effect of stress and anxiety associated with maternal prenatal diagnosis by invasive methods, a study by Allison et al. [18] evaluated 200 pregnant women using the Beck Anxiety Inventory and Maternal or Paternal Antenatal Attachment Scales. It was observed that the anxiety levels of women who had had an invasive test were greater than those of women at booking and after an abnormality scan. In addition, from the time of booking until the time of the anomaly scan, fear decreased while attachment developed. In women who had undergone an invasive test, there was an association between anxiety and attachment, similarly to the levels of maternal–fetal attachment identified in the current study, although it was not determined what category of pregnant women at risk developed the highest attachment levels. A similar finding of pre-procedural high anxiety levels was observed in a prospective study of 232 pregnant women carrying a pregnancy at risk for genetic abnormalities, and they also manifested solid maternal–fetal attachment [31].

Other studies evaluated the influence of noninvasive prenatal diagnosis tests (NIPD) on the psychology and attitudes of the pregnant women involved, as opposed to the present study that assessed them before undergoing an invasive diagnostic test. The fact that the NIPD is a noninvasive test may alleviate a parent’s worry of causing damage to the fetus during an invasive treatment; however, the anxiety and anguish often associated with a diagnostic predicament still persist. One study highlighted the dread of hearing negative news as the primary reason patients experience anxiety before invasive procedures [32,33,34]. In addition, they discovered that patients who received information from physicians or nurses had lower anxiety levels compared to those who received no information or information from friends and relatives. The fact that the NIPD is only accessible in a regulated medical setting (and cannot be purchased online) may assist in ensuring high-quality counseling and alleviate patients’ concerns. In noninvasive prenatal screening circumstances other than NIPD, offering accurate information about testing and assisting patients in making educated choices were reported to lessen anxiety [35]. However, the current study did not evaluate post-test psychological problems faced by pregnant women who received an intervention about stressful situations before the diagnostic test in comparison to those who did not.

The acknowledgment of having a child with potential genetic disorders or malformations is not only experienced by the CFTR-carrier women, who represented the main focus of the study, but also by those who acknowledge that their pregnancy is at risk and require invasive diagnostic procedures. For example, women older than 35 have the status of advanced maternal age that is connected with certain pregnancy-related hazards. Being “at risk” produces anxiety and worry, which older pregnant women attempt to alleviate by gaining as much knowledge as possible, which is daunting to some women owing to the abundance of accessible information [36]. Oppositely, there can be a certain level of higher social reluctance and lower social acceptance towards these particular cases. Nevertheless, CFTR-carrier pregnant women did not score significantly different results in the Prenatal Psychosocial Profile regarding stress levels, social support, and self-esteem, probably indicating that the acknowledgement of having a child with potential genetic disorders or malformations does not determine significant changes until or after birth, as other studies suggest [37].

In addition to the stress faced by women undergoing invasive diagnosis procedures, others experience high levels of stress due to infertility reasons. A vast amount of research has documented the challenges faced by infertile couples and the stress connected with the different phases of the assisted reproductive methods journey, demonstrating the need for counseling [38,39]. These couples manifested high levels of anxiety over the health of their newborns when they faced infertility issues. The same couples had more intimacy and consensus problems compared to couples that were not at risk of having a child with a mental or physical disability. Similar findings were observed in couples who were taking care of a child with disabilities [40], although there were no findings that assessed the marital attitudes for couples at risk of having a child with potential genetic disorders or malformations before the invasive diagnostics test, as the current study does.

Regarding the questionnaires completed by the participants in this study, the updated PRAQ-R2 has been shown to be adequately suited for use in pregnant women regardless of parity since it regularly tests similar constructs throughout pregnancy. Future investigations assessing pregnancy-specific anxiety using the PRAQ-R2 will be able to evaluate and aggregate the scores of primiparous and multiparous pregnant women with more ease if one of the questions is reworded as suggested. Better reference scores and materials for all pregnant women will further facilitate screening of pregnant women at particular risk for developing high levels of anxiety and may demonstrate useful for child development, allowing for the allocation of appropriate prevention and intervention programs for pregnant women [41].

The Prenatal Attachment Inventory (PAI) is one of the most often used surveys for assessing maternal–fetal bonding and is assessed based on five subscales: affect, interaction, differentiation, fantasy, and sensitivity. In this study, symptoms of depression were greater than those described in prior investigations of normal pregnant women. Maternal adjustment and gestational age raised maternal–fetal attachment (MFA) substantially. Affect and interaction were the MFA aspects most impacted by gestational age, while maternal–fetal differentiation was related to gestational age and maternal adjustment [42]. There was a correlation between depressive symptoms and an increase in fantasy and sensitivity component scores. Prenatal attachment grew as gestational age progressed and mothers felt higher degrees of pair adjustment, promoting MFA strength, good affect, and MFA interaction and differentiation [43].

### 4.2. Strengths and Limitations

Although the current study satisfied the minimum requirements for sample size for both study groups, there are several limitations that should be mentioned. First, the cross-sectional design can be regarded as a limiting factor as it does not allow for a dynamic evaluation and assessment in time of the stress levels. Second, the use of questionnaires can have a high subjectivity index from all participants who agree to complete them, which might result in biases. Lastly, the conclusions of the current study are limited to the studied population since country-specific features and cultural particularities in Romania could impact the results.

## 5. Conclusions

The presented findings indicate that CFTR-carrier mothers are more affectionate to the fetus, with better maternal–fetal quality and intensity of attachment compared to other pregnant women with at-risk pregnancies. However, couple intimacy and consensus problems were more often experienced by CFTR carriers. Based on the current findings, it is likely that CFTR-carrier mothers have a better perception of the possible pregnancy outcomes by knowing their abnormal gene carrier status. Therefore, the psychological impact of invasive diagnostic tests is lower in this category compared with those who are unaware of the possible pregnancy outcomes. Nonetheless, it should be promoted that pregnant women with a moderate risk of giving birth to a child with CF or other congenital malformations should undergo noninvasive prenatal diagnosis (NIPD) tests that become enabled by new technologies and better precision rates. It is expected that a safe test such as the NIPD will become more popular among pregnant women and uptake will be high, decreasing the stress of undertaking prenatal testing, and the stress associated with expecting an invasive diagnostic procedure.

## Figures and Tables

**Table 1:** Comparison of background characteristics between CFTR-carrier and non-carrier pregnant women.

Variables	Carriers (*n* = 84)	Non-Carriers (*n* = 91)	*p*-Value
Age range (years)			0.002
<25	27 (32.1%)	16 (17.6%)	
25–35	39 (46.4%)	34 (37.4%)	
>35	18 (21.4%)	41 (45.1%)	
**Area of Residence**			0.531
Rural	33 (39.3%)	40 (44.0%)	
Urban	51 (60.7%)	51 (56.0%)	
**Relationship Status**			0.649
Married/Concubinage	79 (94.0%)	84 (92.3%)	
Single/Divorced/Widowed	5 (6.0%)	7 (7.7%)	
**Income**			0.683
Low	16 (19.0%)	14 (15.4%)	
Medium	43 (51.2%)	45 (49.5%)	
High	25 (29.8%)	32 (35.2%)	
**Education**			0.830
Primary education	6 (7.1%)	5 (5.5%)	
High school	24 (28.6%)	29 (31.9%)	
Higher education	54 (64.3%)	57 (62.6%)	
**Occupation**			0.613
Employed/Self-Employed	69 (82.1%)	72 (79.1%)	
Unemployed	15 (17.9%)	19 (20.9%)	
**Sexual activity**			0.265
Weekly or more	29 (34.5%)	22 (24.2%)	
1–3 times per month	44 (52.4%)	51 (56.0%)	
Less than 1–3 times per month	11 (13.1%)	18 (19.8%)	
**Behavior**			
Frequent alcohol consumption	1 (1.2%)	3 (3.3%)	0.351
Frequent smoker	14 (16.7%)	12 (13.2%)	0.517

Data reported as *n* (frequency) and calculated using chi-square test and Fisher’s exact unless specified differently.

**Table 2:** Comparison of obstetrical characteristics and comorbid conditions between CFTR-carrier and non-carrier pregnant women.

Variables	Carriers (*n* = 84)	Non-Carriers (*n* = 91)	*p*-Value
**Gravidity**			0.045
1	63 (75.0%)	56 (61.5%)	
2	16 (19.0%)	19 (20.9%)	
≥3	5 (6.0%)	16 (17.6%)	
**Parity**			0.292
**0**	80 (95.2%)	83 (91.2%)	
≥1	4 (4.8%)	8 (8.8%)	
**Type of evaluation planned**			0.447
CVS	38 (45.2%)	36 (39.6%)	
Amniocentesis	46 (54.8%)	55 (60.4%)	
Pregnancy-associated complications **			0.693
0	67 (85.7%)	79 (86.8%)	
1	8 (9.5%)	6 (6.6%)	
≥2	4 (4.8%)	6 (6.6%)	
Body mass index ***			0.609
Normal weight	64 (76.2%)	68 (74.7%)	
Overweight	12 (14.3%)	17 (18.7%)	
Obese	8 (9.5%)	6 (6.6%)	
**History of pregnancy loss**			0.340
None	4 (79.3%)	6 (6.6%)	
Medical abortion	3 (3.3%)	4 (4.4%)	
Stillbirth (>20 weeks)	40 (7.1%)	31 (34.1%)	
Miscarriage (<20 weeks)	37 (10.3%)	50 (54.9%)	
**Comorbidities**			
Cardiovascular	6 (7.1%)	8 (8.8%)	0.688
Metabolic	5 (6.0%)	5 (5.5%)	0.896
Autoimmune	2 (2.4%)	1 (1.1%)	0.513
Respiratory	5 (6.0%)	7 (7.7%)	0.649
Other	3 (3.6%)	3 (3.3%)	0.920
History of depression	6 (7.1%)	8 (8.8%)	0.688
History of COVID-19	9 (10.7%)	7 (7.7%)	0.488

Data reported as *n* (frequency) and calculated using chi-square test and Fisher’s exact unless specified differently; ** including high blood pressure, gestational diabetes, infections, preeclampsia; *** adjusted for the month of pregnancy; CVS—chorionic villus sampling.

**Table 3:** PAI survey results for CFTR-carrier and non-carrier pregnant women.

Items (Score Range)	Carriers (*n* = 84)	Non-Carriers (*n* = 91)	*p*-Value
Affect (6–24)	17.52 ± 4.18	15.32 ± 5.09	0.002
Interaction (5–20)	13.80 ± 4.03	12.94± 4.34	0.177
Differentiation (4–16)	10.94 ± 3.40	9.81 ± 3.56	0.033
Fantasy (3–12)	8.06 ± 3.55	7.57 ± 4.10	0.400
Sensitivity (3–12)	5.27 ± 2.81	6.04 ± 2.16	0.042
Total score (21–84)	57.13 ± 10.28	51.35 ± 9.84	<0.001

Data reported as *n* (frequency) and calculated using chi-square test and Fisher’s exact unless specified differently. PAI—Prenatal Attachment Interview.

**Table 4:** MAAS survey results for CFTR-carrier and non-carrier pregnant women.

Components	Carriers (*n* = 84)	Non-Carriers (*n* = 91)	*p*-Value
Quality of attachment	48.6 ± 11.7	45.3 ± 9.9	0.045
Intensity of attachment	28.3 ± 6.1	23.0 ± 5.7	<0.001
Global attachment score	70.2 ± 19.5	66.2 ± 17.6	0.155

Data reported as *n* (frequency) and calculated using chi-square test and Fisher’s exact unless specified differently. MAAS—Maternal Antenatal Attachment Scale.

**Table 5:** PRAQ-R2 survey results for CFTR-carrier and non-carrier pregnant women.

Subscales	Carriers (*n* = 84)	Non-Carriers (*n* = 91)	*p*-Value
Fear of giving birth (3 to 15)	7.08 ± 2.94	7.91 ± 3.12	0.072
Worries about bearing a physically or mentally handicapped child (4 to 20)	11.35 ± 4.33	12.84 ± 5.30	0.011
Concern about one’s own appearance (3 to 15)	5.19 ± 1.86	5.37 ± 1.92	0.530
Total scale (10 to 50)	23.62 ± 7.11	26.12 ± 8.08	0.031

Data reported as *n* (frequency) and calculated using chi-square test and Fisher’s exact unless specified differently. PRAQ-R2—Pregnancy-Related Anxiety Questionnaire.

**Table 6:** PPP survey results for CFTR-carrier and non-carrier pregnant women.

Subscales	Carriers (*n* = 84)	Non-Carriers (*n* = 91)	*p*-Value
Stress	27.14 ± 4.31	26.88 ± 5.33	0.724
Social support from partner	51.93 ± 11.82	49.43 ± 10.71	0.144
Social support from other people	38.16 ± 7.08	39.26 ± 8.21	0.345
Self-esteem	23.48 ± 4.26	24.37 ± 5.66	0.244

Data reported as *n* (frequency) and calculated using chi-square test and Fisher’s exact unless specified differently. PPP—Prenatal Psychosocial Profile.

**Table 7:** MIQ survey results for CFTR-carrier and non-carrier pregnant women.

Variables	Carriers (*n* = 84)	Non-Carriers (*n* = 91)	*p*-Value
Intimacy problems	38.15 ± 10.38	34.38 ± 8.62	0.009
Consensus	35.43 ± 7.92	33.06 ± 7.19	0.039
Openness	37.06 ± 7.37	38.13 ± 8.78	0.385
Affection	27.61 ± 5.29	30.28 ± 6.06	0.002
Commitment	31.59 ± 6.34	30.77 ± 5.94	0.378

Data reported as *n* (frequency) and calculated using chi-square test and Fisher’s exact unless specified differently. MIQ—Marital Intimacy Questionnaire.

## Data Availability

The data presented in this study are available on request from the corresponding author.
